# Genome sequence of the clover-nodulating *Rhizobium leguminosarum* bv. *trifolii* strain SRDI943.

**DOI:** 10.4056/sigs.4478252

**Published:** 2013-12-15

**Authors:** Wayne Reeve, Elizabeth Drew, Ross Ballard, Vanessa Melino, Rui Tian, Sofie De Meyer, Lambert Brau, Mohamed Ninawi, Hajnalka Daligault, Karen Davenport, Tracy Erkkila, Lynne Goodwin, Wei Gu, Christine Munk, Hazuki Teshima, Yan Xu, Patrick Chain, Nikos Kyrpides

**Affiliations:** 1 Centre for Rhizobium Studies, Murdoch University, Western Australia, Australia; 2 South Australian Research and Development Institute, Urrbrae, South Australia, Australia; 3 School of Life and Environmental Sciences, Faculty of Science & Technology, Deakin University, Melbourne, Victoria, Australia; 4 Los Alamos National Laboratory, Bioscience Division, Los Alamos, New Mexico, USA; 5 DOE Joint Genome Institute, Walnut Creek, California, USA

**Keywords:** root-nodule bacteria, nitrogen fixation, rhizobia, *Alphaproteobacteria*

## Abstract

*Rhizobium leguminosarum* bv. *trifolii* SRDI943 (strain syn. V2-2) is an aerobic, motile, Gram-negative, non-spore-forming rod that was isolated from a root nodule of *Trifolium michelianum* Savi cv. Paradana that had been grown in soil collected from a mixed pasture in Victoria, Australia. This isolate was found to have a broad clover host range but was sub-optimal for nitrogen fixation with *T. subterraneum* (fixing 20-54% of reference inoculant strain WSM1325) and was found to be totally ineffective with the clover species *T. polymorphum* and *T. pratense*. Here we describe the features of *R. leguminosarum* bv. *trifolii* strain SRDI943, together with genome sequence information and annotation. The 7,412,387 bp high-quality-draft genome is arranged into 5 scaffolds of 5 contigs, contains 7,317 protein-coding genes and 89 RNA-only encoding genes, and is one of 100 rhizobial genomes sequenced as part of the DOE Joint Genome Institute 2010 Genomic Encyclopedia for Bacteria and Archaea-Root Nodule Bacteria (GEBA-RNB) project.

## Introduction

The availability of usable nitrogen (N) is vital for productivity in agricultural systems that are N-deficient [1]. It can be supplied exogenously in the form of industrially synthesized fertilizers. However, this practice is expensive since fertilizer manufacture depends on the availability of fossil fuels that are burnt to support the industrial process of chemical N-fixation. A far more economical practice is to supply plant-available N to farming systems by exploiting the process of biological N-fixation that occurs in a symbiotic relationship between legumes and their rhizobial microsymbionts [2]. In this specific association, atmospheric inert dinitrogen gas is converted into bioavailable N to support legume growth.

Pasture legumes, including the clovers that comprise the *Trifolium* genus, are major contributors of biologically fixed nitrogen (N_2_) to mixed farming systems throughout the world [3,4]. In Australia, soils with a history of growing *Trifolium* spp. have developed large and symbiotically diverse populations of *Rhizobium leguminosarum* bv. *trifolii* (*R. l. trifolii*) that are able to infect and nodulate a range of clover species. The N_2_-fixation capacity of the symbioses established by different combinations of clover hosts (*Trifolium* spp.) and strains of *R. l. trifolii* can vary from 10 to 130% when compared to an effective host-strain combination [5-8].

*R. l. trifolii* strain SRDI943 (syn. V2-2 [9]) was isolated from a nodule recovered from the roots of the annual clover *Trifolium michelianum* Savi cv. Paradana that had been inoculated with soil collected from under a mixed pasture at Walpeup, Victoria, Australia and grown in N deficient media for four weeks after inoculation, in the greenhouse [10]. SRDI943 forms an effective symbiosis with *T. purpureum* but sub-optimal N_2_-fixation symbiosis with *T. subterraneum* cv. Campeda and Clare (~24 and 54% respectively of that with strain WSM1325 [9,11]). Here we present a preliminary description of the general features for *R. l. trifolii* strain SRDI943 together with its genome sequence and annotation.

## Classification and general features

**Figure 1:**
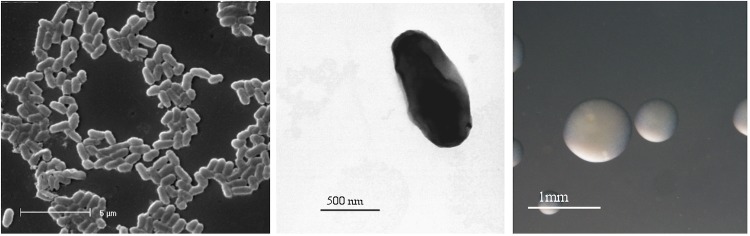
Images of *Rhizobium leguminosarum* bv. *trifolii* strain SRDI943 using scanning (Left) and transmission (Center) electron microscopy as well as light microscopy to show the colony morphology on solid media (Right).

*R. l. trifolii* strain SRDI943 is a motile, Gram-negative rod (Figure 1 Left and Center) in the order *Rhizobiales* of the class *Alphaproteobacteria*. It is fast growing, forming colonies within 3-4 days when grown on half strength Lupin Agar (½LA) [12] at 28°C. Colonies on ½LA are white-opaque, slightly domed and moderately mucoid with smooth margins (Figure 1 Right).

**Figure 2:**
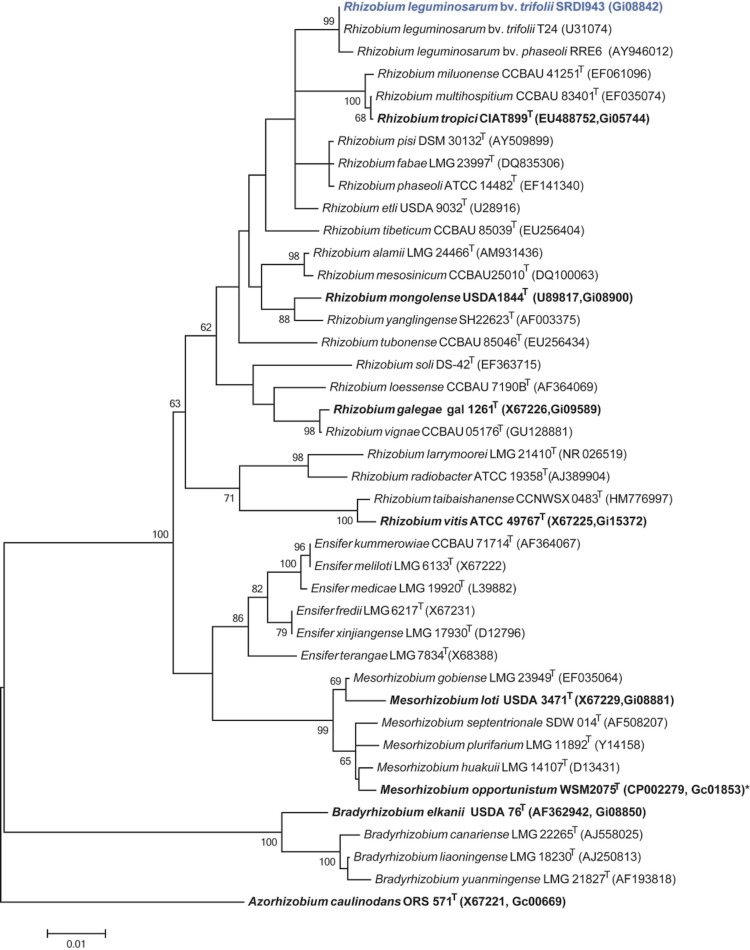
Phylogenetic tree showing the relationship of *Rhizobium leguminosarum* bv. *trifolii* SRDI943 (shown in blue print) with some of the root nodule bacteria in the order *Rhizobiales* based on aligned sequences of the 16S rRNA gene (1,307 bp internal region). All sites were informative and there were no gap-containing sites. Phylogenetic analyses were performed using MEGA, version 5.05 [31]. The tree was built using the maximum likelihood method with the General Time Reversible model. Bootstrap analysis [32] with 500 replicates was performed to assess the support of the clusters. Type strains are indicated with a superscript T. Strains with a genome sequencing project registered in GOLD [33] are in bold print and the GOLD ID is mentioned after the accession number. Published genomes are indicated with an asterisk.

Minimum information about the Genome Sequence (MIGS) is provided in Table 1. Figure 2 shows the phylogenetic relationship of *R. l. trifolii* strain SRDI943 to root nodule bacteria in the order Rhizobiales in a 16S rRNA sequence based tree. This strain clusters closest to *R. l. trifolii* T24 and *Rhizobium leguminosarum* bv. *phaseoli* RRE6 with 100% and 99.8% sequence identity, respectively.

**Table 1:** Classification and general features of *Rhizobium leguminosarum* bv. *trifolii* SRDI943 according to the MIGS recommendations [13]

**MIGS ID**	**Property**	**Term**	**Evidence code**
	Current classification	Domain *Bacteria*	TAS [14]
		Phylum *Proteobacteria*	TAS [15]
		Class *Alphaproteobacteria*	TAS [16,17]
		Order *Rhizobiales*	TAS [17,18]
		Family *Rhizobiaceae*	TAS [19-21]
		Genus *Rhizobium*	TAS [21-26]
		Species *Rhizobium leguminosarum* bv. *trifolii*	TAS [21,23,27,28]
	
	Gram stain	Negative	IDA
	Cell shape	Rod	IDA
	Motility	Motile	IDA
	Sporulation	Non-sporulating	NAS
	Temperature range	Mesophile	NAS
	Optimum temperature	28°C	NAS
	Salinity	Non-halophile	NAS
MIGS-22	Oxygen requirement	Aerobic	TAS [11]
	Carbon source	Varied	NAS
	Energy source	Chemoorganotroph	NAS
MIGS-6	Habitat	Soil, root nodule, on host	TAS [9]
MIGS-15	Biotic relationship	Free living, symbiotic	TAS [9]
MIGS-14	Pathogenicity	Non-pathogenic	NAS
	Biosafety level	1	TAS [29]
	Isolation	Root nodule	TAS [9]
MIGS-4	Geographic location	Victoria, Australia	TAS [9]
MIGS-5	Soil collection date	Dec, 1998	IDA
MIGS-4.1 MIGS-4.2	Longitude Latitude	142.0262 -35.13531	IDA
MIGS-4.3	Depth	0-10cm	
MIGS-4.4	Altitude	Not recorded	

Evidence codes – IDA: Inferred from Direct Assay; TAS: Traceable Author Statement (i.e., a direct report exists in the literature); NAS: Non-traceable Author Statement (i.e., not directly observed for the living, isolated sample, but based on a generally accepted property for the species, or anecdotal evidence). These evidence codes are from the Gene Ontology project [30].

## Symbiotaxonomy

*R. l. trifolii* SRDI943 forms nodules on (Nod^+^) and fixes N_2_ (Fix^+^) with a range of annual and perennial clover species of Mediterranean origin (Table 2). SRDI943 forms white, ineffective (Fix^-^) nodules with the perennial clover *T. pratense* and *T. polymorphum*.

**Table 2:** Compatibility of SRDI943 with eleven *Trifolium* genotypes for nodulation (Nod) and N_2_-Fixation (Fix)

**Species Name**	**Cultivar**	**Common Name**	**Growth Type**	**Nod**	**Fix**	**Reference**
*T. glanduliferum* Boiss.	Prima	Gland	Annual	+	+	
*T. michelianum* Savi.	Bolta	Balansa	Annual	+	+	
*T. purpureum* Loisel	Paratta	Purple	Annual	+	+	[11]
*T. resupinatum* L.	Kyambro	Persian	Annual	+	+	
*T. subterraneum* L.	Campeda	Sub. clover	Annual	+	+	[9,11]
*T. subterraneum* L.	Clare	Sub. clover	Annual	+	+	[9,11]
*T. vesiculosum* Savi.	Arrotas	Arrowleaf	Annual	+	+	
*T. fragiferum* L.	Palestine	Strawberry	Perennial	+	+	
*T. polymorphum* Poir	Acc.#087102	Polymorphous	Perennial	+(w)	-	[11]
*T. pratense* L.	-	Red	Perennial	+(w)	-	
*T. repens* L.	Haifa	White	Perennial	+	+	

(w) indicates nodules present were white.

## Genome sequencing and annotation information

### Genome project history

This organism was selected for sequencing on the basis of its environmental and agricultural relevance to issues in global carbon cycling, alternative energy production, and biogeochemical importance, and is part of the Community Sequencing Program at the U.S. Department of Energy, Joint Genome Institute (JGI) for projects of relevance to agency missions. The genome sequence is deposited in the Genomes OnLine Database (GOLD) [33] and an improved-high-quality-draft genome sequence in IMG/GEBA. Sequencing, finishing and annotation were performed by the JGI. A summary of the project information is shown in Table 3.

**Table 3:** Genome sequencing project information for *Rhizobium leguminosarum* bv. *trifolii* strain SRDI943.

**MIGS ID**	**Property**	**Term**
MIGS-31	Finishing quality	Improved high-quality draft
MIGS-28	Libraries used	2× Illumina libraries; Std short PE & CLIP long PE
MIGS-29	Sequencing platforms	Illumina HiSeq 2000
MIGS-31.2	Sequencing coverage	Illumina (761×)
MIGS-30	Assemblers	Velvet 1.1.05, phrap SPS-4.24, Allpaths version 39750
MIGS-32	Gene calling methods	Prodigal 1.4, GenePRIMP
	GOLD ID	Gi08842
	NCBI project ID	89687
	Database: IMG	2517093000
	Project relevance	Symbiotic N_2_ fixation, agriculture

### Growth conditions and DNA isolation

*R. l. trifolii* strain SRDI943 was cultured to mid logarithmic phase in 60 ml of TY rich media [34] on a gyratory shaker at 28°C. DNA was isolated from the cells using a CTAB (Cetyl trimethyl ammonium bromide) bacterial genomic DNA isolation method [35].

### Genome sequencing and assembly

The genome of *R. l. trifolii* strain SRDI943 was sequenced at the Joint Genome Institute (JGI) using an Illumina sequencing platform. An Illumina short-insert paired-end (PE) library with an average insert size of 270 bp produced 18,764,470 reads and an Illumina CLIP long-insert paired-end (PE) library with an average insert size of 9,482 bp produced 18,761,080 reads totaling 5,629 Mb of Illumina data for this genome. All general aspects of library construction and sequencing performed at the JGI can be found at the DOE JGI user homepage [35]. The initial draft assembly contained 5 contigs in 5 scaffolds. The initial draft data was assembled with Allpaths, version 39750. The Allpaths consensus was computationally shredded into 10 Kb overlapping fake reads (shreds). Illumina sequencing data were assembled with Velvet, version 1.1.05 [36], and the consensus sequences were computationally shredded into 1.5 kb overlapping fake reads (shreds). The Allpaths consensus shreds, the Illumina VELVET consensus shreds and a sub-set of the Illumina CLIP paired-end reads were integrated using parallel phrap, version SPS - 4.24 (High Performance Software, LLC). The software Consed [37-39] was used in the following finishing process. The estimated genome size is 7.4 Mb and the final assembly is based on 5,629 Mb of Illumina draft data which provides an average of 761× coverage of the genome.

### Genome annotation

Genes were identified using Prodigal [40] as part of the DOE-JGI annotation pipeline [41] annotation pipeline, followed by a round of manual curation using the JGI GenePRIMP pipeline [42]. The predicted CDSs were translated and used to search the National Center for Biotechnology Information (NCBI) non-redundant database, UniProt, TIGRFam, Pfam, PRIAM, KEGG, COG, and InterPro databases. These data sources were combined to ascribe a product description for each predicted protein. Non-coding genes and miscellaneous features were predicted using tRNAscan-SE [43], RNAMMer [44], Rfam [45], TMHMM [46], and SignalP [47]. Additional gene prediction analyses and functional annotation were performed within the Integrated Microbial Genomes (IMG-ER) platform [35,48].

## Genome properties

**Figure 3:**
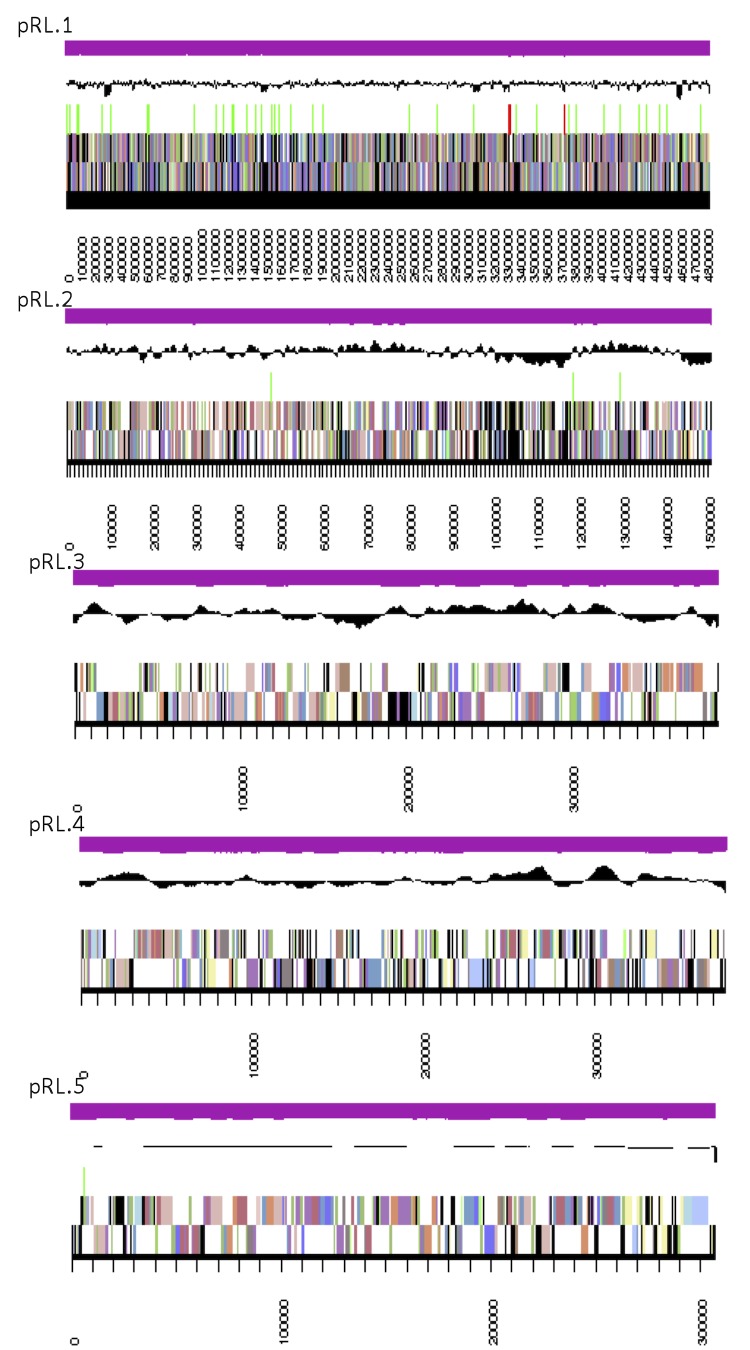
Graphical map of the genome of *Rhizobium leguminosarum* bv. *trifolii* strain SRDI943. From bottom to the top of each scaffold: Genes on forward strand (color by COG categories as denoted by the IMG platform), Genes on reverse strand (color by COG categories), RNA genes (tRNAs green, sRNAs red, other RNAs black), GC content, GC skew.

The genome is 7,412,387 nucleotides with 60.69% GC content (Table 4) and comprised of 5 scaffolds (Figure 3) of 5 contigs. From a total of 7,406 genes, 7,317 were protein encoding and 89 RNA only encoding genes. The majority of genes (78.5%) were assigned a putative function whilst the remaining genes were annotated as hypothetical. The distribution of genes into COGs functional categories is presented in Table 5.

**Table 4:** Genome Statistics for *Rhizobium leguminosarum* bv. *trifolii* SRDI943

**Attribute**	**Value**	**% of Total**
Genome size (bp)	7,412,387	100.00
DNA coding region (bp)	6,395,342	86.28
DNA G+C content (bp)	4,498,817	60.69
Number of scaffolds	5	
Number of contigs	5	
Total gene	7,406	100.00
RNA genes	89	1.20
rRNA operons	3	
Protein-coding genes	7,317	98.80
Genes with function prediction	5,814	78.50
Genes assigned to COGs	5,770	77.91
Genes assigned Pfam domains	6,032	81.45
Genes with signal peptides	631	8.52
Genes with transmembrane proteins	1,618	21.85
CRISPR repeats	0	

**Table 5:** Number of protein coding genes of *Rhizobium leguminosarum* bv. *trifolii* SRDI943 associated with the general COG functional categories.

**Code**	**Value**	**%age**	**COG Category**
J	196	3.03	Translation, ribosomal structure and biogenesis
A	1	0.02	RNA processing and modification
K	652	10.06	Transcription
L	231	3.57	Replication, recombination and repair
B	2	0.03	Chromatin structure and dynamics
D	40	0.62	Cell cycle control, mitosis and meiosis
Y	0	0.00	Nuclear structure
V	76	1.17	Defense mechanisms
T	373	5.76	Signal transduction mechanisms
M	334	5.16	Cell wall/membrane biogenesis
N	92	1.42	Cell motility
Z	1	0.02	Cytoskeleton
W	1	0.02	Extracellular structures
U	95	1.47	Intracellular trafficking and secretion
O	193	2.98	Posttranslational modification, protein turnover, chaperones
C	324	5.00	Energy production conversion
G	714	11.02	Carbohydrate transport and metabolism
E	659	10.17	Amino acid transport metabolism
F	109	1.68	Nucleotide transport and metabolism
H	192	2.96	Coenzyme transport and metabolism
I	227	3.50	Lipid transport and metabolism
P	333	5.14	Inorganic ion transport and metabolism
Q	165	2.55	Secondary metabolite biosynthesis, transport and catabolism
R	842	13.00	General function prediction only
S	627	9.68	Function unknown
-	1,636	22.09	Not in COGS
